# Beyond Blood Sugar: Low Awareness of Kidney Disease among Type 2 Diabetes Mellitus Patients in Dalmatia—Insights from the First Open Public Call

**DOI:** 10.3390/medicina60101643

**Published:** 2024-10-08

**Authors:** Josipa Radić, Marijana Vučković, Hana Đogaš, Marina Grubić, Andrej Belančić, Leida Tandara, Lucija Šolić Šegvić, Ivana Novak, Mislav Radić

**Affiliations:** 1 Department of Internal Medicine, Division of Nephrology and Dialysis, University Hospital of Split, 21000 Split, Croatia; josiparadic1973@gmail.com (J.R.); mavuckovic@kbsplit.hr (M.V.); lucijasolicc@gmail.com (L.Š.Š.); ivana.i.novak@gmail.com (I.N.); 2 Internal Medicine Department, School of Medicine, University of Split, 21000 Split, Croatia; 3 School of Medicine, University of Split, 21000 Split, Croatia; hana.dogas@gmail.com; 4 Institute for Emergency Medicine of Split-Dalmatia County, 21000 Split, Croatia; marina.grubic123@gmail.com; 5 Department of Basic and Clinical Pharmacology with Toxicology, Faculty of Medicine, University of Rijeka, Braće Branchetta 20, 51000 Rijeka, Croatia; andrej.belancic@uniri.hr; 6 Division of Medical Laboratory Diagnostic, University Hospital of Split, 21000 Split, Croatia; leida.tandara@gmail.com; 7 Department of Internal Medicine, Division of Rheumatology, Allergology and Clinical Immunology, University Hospital of Split, 21000 Split, Croatia

**Keywords:** diabetes, kidney disease, awareness, obesity, Mediterranean diet

## Abstract

*Background and Objectives*: Kidney disease (KD) is a common complication of diabetes mellitus (DM) associated with adverse outcomes of renal failure, cardiovascular disease, and mortality. The aim of this study was to determine the prevalence and awareness of the KD among the DM type 2 (T2DM) patients. *Materials and Methods*: This cross-sectional study was conducted at the University Hospital of Split between November and December of 2023 during an open call for DM patients. For each participant, blood and urine samples, along with relevant medical information, were collected, and adherence to the Mediterranean diet (MeDi) was assessed using the Mediterranean Diet Service Score (MDSS). Furthermore, blood pressure was measured, along with body composition and anthropometric parameters. *Results*: Of 252 T2DM patients with a median age of 67 years (IQR: 60–73), 130 (51.6%) were women. The median duration of T2DM was 10 years (IQR: 6–20). Despite the fact that 80.95% of total participants reported receiving dietary guidelines from any source, only 53.2% reported adhering to the suggested instructions, while according to the MDSS, only 7.2% adhered to the MeDi. The median body mass index was 27.6 kg/m^2^ (24.2–31), with 70.1% of participants overweight or obese. Only 6% of participants believed they had KD, but after blood and urine sample analysis, 31% were found to have KD. *Conclusions*: This study highlights a significant gap in awareness of KD, low adherence to MeDi, and a high prevalence of obesity among T2DM patients. Due to the increasing number of T2DM patients, it is crucial to improve healthy lifestyle education and make modifications within this group, as well as perform regular screening for KD and medical check-ups.

## 1. Introduction

Type 2 diabetes mellitus (T2DM) is a chronic metabolic disease characterized by insulin resistance and impaired glucose metabolism, with an alarming increase in global prevalence predicted for the coming decades [1,2]. The risk factors for T2DM, including genetic predisposition, physical inactivity, and poor dietary habits, are exacerbated by the increasing prevalence of obesity, which is fueling the sharp rise in T2DM rates worldwide [1,3,4]. Adequate glucose regulation and careful selection of pharmacotherapy, taking into the account not only glycemic control but also major adverse cardiovascular events (MACEs), renal protection, and anti-obesogenic effects is crucial to prevent a wide range of complications. Among these complications, renal complications are particularly important, with chronic kidney disease (CKD) being a common and serious consequence of T2DM [3,5,6,7].

The global financial burden of diabetes is substantial and projected to rise dramatically. In 2021, healthcare expenditures related to diabetes reached a staggering USD 966 billion worldwide and are expected to exceed USD 1054 billion by 2045 [4]. By 2030, the economic impact of diabetes and its complications is estimated to reach USD 2.1 trillion, representing a 61% increase from 2015 [8]. To compare, according to data from the Croatian Society for Pharmacoeconomics and Health Economics (2016), approximately HRK 4.6 billion (Croatian Kuna) was spent on the treatment of type 2 diabetes in a single year in Croatia. This accounted for 19.8% of the total budget of the Croatian Health Insurance Fund. A majority of these expenditures (88.1%) were allocated to the treatment of diabetes-related complications, while smaller portions were spent on pharmacotherapy (8.1%), medical supplies (2.5%), and patient monitoring and basic care across all levels of the healthcare system (1.3%) [9]. The national financial burden is expected to follow global trends, with projections for Croatia indicating an increase in the prevalence of T2DM. The number of people living with T2DM is projected to rise from 212.7 thousand out of 3.06 million (6.95%) to 193.9 thousand out of 2.56 million (7.57%) until 2045 [10].

Beyond the economic impact, the increasing prevalence of T2DM is a serious public health concern due to its severe implications for overall quality of life. In 2017, T2DM was ranked as the seventh leading cause of Disability-Adjusted Life Years (DALYs), highlighting the profound impact of the condition on individuals’ well-being and longevity [11,12]. CKD affects around 40% of diabetic individuals, resulting in a significant clinical and economic burden due to its progressive nature and accompanying comorbidities [13,14]. Individuals with diabetes and CKD are at risk for acute and long-term complications, kidney failure, and cardiovascular complications. Comprehensive diabetes care includes regular screening for these complications and managing cardiovascular risk factors, including hypertension; dyslipidemia; obesity; and lifestyle factors, like diet, smoking, and physical activity. Regular monitoring and evaluation are crucial for effective diabetes management [15].

Public awareness regarding diabetes, and especially its complications, like CKD, remains inadequate, with limited knowledge on the importance of regular monitoring of kidney function and laboratory parameters [16,17]. These issues are exacerbated in Croatia’s Dalmatia region by a high prevalence of obesity and poor adherence to the Mediterranean diet (MeDi), which is known for its preventive effects against cardiovascular and renal outcomes [18,19,20,21]. This publication seeks to study the existing awareness of kidney disease (KD), dietary adherence, and prevalence of obesity among T2DM patients in Dalmatia, as well as suggest potential for enhancing patient education, clinical practice, and public health.

## 2. Materials and Methods

### 2.1. Study Design and Ethical Considerations

This research was conducted as a cross-sectional study and was carried out at the Division of Medical Laboratory Diagnostic, University Hospital of Split and Division of Nephrology and Dialysis, Department of Internal Medicine, University Hospital Centre Split, Croatia, between November and December 2023 (month of World Diabetes Day), during an open public call for diabetic patients. The study protocol was accepted by the Ethics Committee of University Hospital of Split on 27 November 2023 (Number, 2181-147/01/06/LJ.7.-23-02; Class, 500-03/23-01/225.). The researchers informed all participants about the purpose and procedures of the study, and from all participants, informed and written consent was obtained.

### 2.2. Subjects

**Figure 1:**
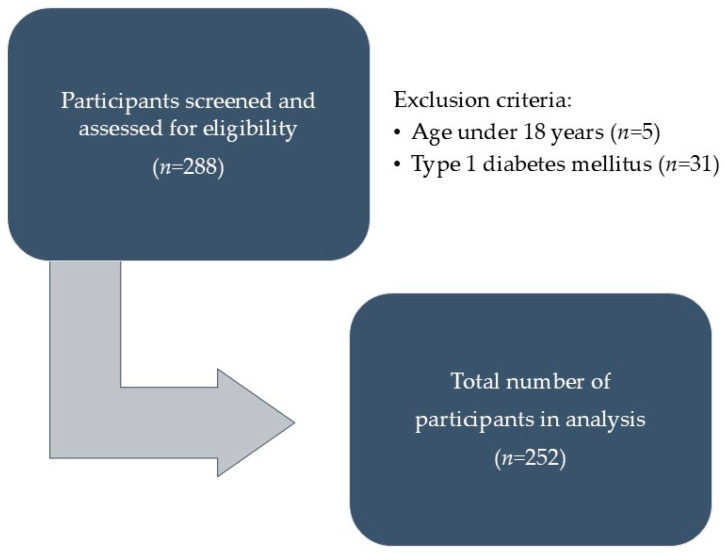
Participant selection process.

Initially, 288 participants were screened during this public call, with minors under parental supervision throughout the screening process. However, five participants were excluded due to being under 18 years of age, and 36 were excluded due to having type 1 DM (of which 5 were under 18 years and 31 were over 18 years). The inclusion criteria required participants to have an existing diagnosis of T2DM. Exclusion criteria included being under 18 years of age or having a diagnosis of type 1 DM. Therefore, a total of 252 participants (130 women and 122 men) with T2DM were included in this study. All participants had been previously diagnosed with T2DM by a family medicine doctor or endocrinologist, in accordance with the criteria of the American Diabetic Association [22]. The study inclusion process is shown in Figure 1.

Relevant medical information was taken by checking participants’ available medical documentation and anamnesis. To assess adherence to MeDi, a semiquantitative food frequency questionnaire called the Mediterranean Diet Serving Score (MDSS) questionnaire was used, as well as survey of their life habits. Moreover, all participants underwent measurements of relevant anthropometric features and body mass composition measurement, blood and urine sampling for laboratory diagnostics, and blood pressure measurement.

### 2.3. Body Composition, Anthropometric, and Blood Pressure Measurements

For each study participant, body composition was assessed using the MC-780 Multi Frequency Segmental Body Mass Analyzer (Tanita, Tokyo, Japan) [23]. This device utilizes bioelectrical impedance analysis technology, which involves sending an imperceptible current through the body to measure the resistance of various tissues, using eight electrodes. This method estimates several body composition parameters, including body mass (kg), percentage of muscle mass (PMM; %), fat-free mass (FFM; kg), fat mass (FM; % and kg), visceral fat (VF) level, and phase angle (PhA; ◦).

Participants with an implanted pacemaker or cardioverter defibrillator were excluded from body composition analysis due to precaution, as this measurement could possibly interfere with these devices. Also, participants with amputated lower or upper extremities could not be measured with this device. Furthermore, all study participants followed specific pre-measurement instructions from the device manual: abstain from eating or drinking for at least 3 h before the measurement; urinate just before the measurement; and avoid alcohol consumption, excessive eating or drinking, and intense exercise for at least one day prior.

Additionally, a non-stretchable, flexible measuring tape was used to determine the circumference of the mid-upper arm (MUAC), waist (WC), and hip (HC). The upper arm circumference was measured with the arm relaxed and extended, and the tape placed horizontally 1 cm above the midpoint of the upper arm. WC was measured above the navel while the participant stood upright, and HC was measured around the widest part of the buttocks, with the tape parallel to the floor. Furthermore, the height parameter was determined by a fixed upright measuring device. All participants were measured barefoot with their backs turned toward the measuring device. Body mass index (BMI) was calculated using body mass and height parameters, while waist-to-hip ratio (WHR) was calculated using WC and HC parameters.

Peripheral blood pressure measurements were conducted using an Omron M6 Comfort HEM-7360-E digital sphygmomanometer (Omron, Kyoto, Japan). The appropriately sized cuff was chosen based on the upper arm circumference and positioned correctly. Measurements were taken in a relaxing environment, with participants seated comfortably, back and arm supported, feet flat on the ground, legs uncrossed, and an empty bladder. Blood pressure was measured three times at one-minute intervals, with the average of the last two readings recorded to obtain peripheral systolic and diastolic blood pressure (pSBP and pDBP, respectively) data.

### 2.4. Lifestyle Questionnaire and Mediterranean Diet Serving Score

Physicians, along with medical students and dietitians under supervision of qualified medical practitioners, administered a lifestyle questionnaire that included questions on general participants’ information, dietary habits, and medical history.

General information included gender, age, year of T2DM diagnosis, smoking status (including prior smoking status and smoking duration of current smokers), participants’ prescribed pharmacotherapy, and medical history. All participants were asked if they ever visited a nephrologist and/or endocrinologist, if they ever received nutritional advice, and about their knowledge of existing KD. Participants’ self-assessment of possible KD was later compared to their laboratory parameters.

Adherence to the MeDi pattern was assessed using the self-reported Mediterranean Diet Serving Score (MDSS) questionnaire. This tool evaluates the recommended consumption frequency of fourteen different food items and food groups, as outlined in the MeDi components. The scoring system assigns three points for types of foods consumed at least twice daily or more (cereals, olive oil, vegetables, and fruit). Two points are awarded for daily consumption of dairy products and nuts, and one point for the recommended weekly intake of potatoes (≤3 servings), legumes (≥2 servings), eggs (2–4 servings), poultry (2 servings), red meat (<2 servings), fish (≥2 servings), sweets (≤2 servings), and fermented beverages (one and two glasses a day for females and males, respectively). Intake that is higher or lower than the recommendations for any MeDi component is scored as zero [24].

The MDSS score ranges from zero (0) to twenty-four (24), with a score of 13.5 or higher indicating adherence to the MeDi. This threshold was rounded up to 14 points, as the scoring system requires whole numbers.

### 2.5. Medical History and Clinical and Laboratory Parameters

Data on the length of treatment and therapy for T2DM, as well as data of other coexisting diseases (KD, arterial hypertension (AH), cardiovascular disease (CVD), cerebrovascular disease (CBD), and other chronic diseases), were obtained for each participant from their medical records and through a questionnaire. On the same day that the rest of the study’s measurements were performed, all study participants took the first morning urine sample and underwent usual peripheral blood sampling.

The collected data included the following laboratory parameters: leukocytes (WBC; ×10^9^/L), red blood cell count (RBC; ×10^12^/L), mean corpuscular volume (MCV; fL), hemoglobin (Hb; g/L), mean cellular hemoglobin (MCH; pg), mean cellular hemoglobin concentration (MCHC; g/L), neutrophils (×10^9^/L), lymphocytes (×10^9^/L), monocytes (×10^9^/L), creatinine (μmol/L), glucose (mmol/L), hemoglobin A1c (HbA1c; %), high-density lipoprotein cholesterol (HDL; mmol/L), triglycerides (Tg; mmol/L), total cholesterol (mmol/L), low-density lipoprotein cholesterol (LDL; mmol/L), albuminuria (mg/mmol), eGFR using Chronic Kidney Disease Epidemiology Collaboration CKD-EPI (mL/min/1.73 m^2^), and albumin-to-creatinine ratio (ACR; mg/mmol). Furthermore, KD was defined, after performing laboratory parameters, as eGFR < 60 mL/min/1.73 m^2^ or albuminuria > 3 mg/mmol.

### 2.6. Statistical Analysis

Statistical analyses were performed using the statistical software MedCalc Statistical Software version 18.2.1 (MedCalc Software bvba, Ostend, Belgium) [25] and SPSS (Statistics for Windows, Version 21.0, Armonk, NY, USA, IBM Corp. Released 2013) [26]. The normality of the distribution of numeric variables was tested by the Shapiro–Wilk test. If the data were normally distributed, they were presented with mean and standard deviation (SD), while, in the cases of non-parametric distribution, data were presented with median and interquartile range (IQR). Categorical data were presented as absolute and relative frequencies. The differences between numeric variables in case of deviation from the normal distribution were tested by Kruskal–Wallis, while normally distributed variables were tested by the χ^2^ test. To test the differences in self-reported KD versus present KD in participants, the McNemar–Bowker test was used. To determine the correlations among MDSS, BMI, and eGFR, Spearman’s rank correlation analysis was performed. Finally, a bivariate regression analysis was performed to analyze the association between measured parameters, particularly the adherence to each MeDi component and MDSS with the progression of kidney failure. The results of logistic regression were provided as odds ratios (ORs) with a 95% confidence interval (95% CI). Significance level was set at *p*-value < 0.05.

## 3. Results

The study sample consisted of 252 T2DM participants, comprising 130 (51.6%) women and 122 men (48.4%), with a median age of 67 years (60–73). Detailed demographic and clinical data, including age, sex, blood pressure, visits to endocrinologists and nephrologists, smoking status, therapy, and comorbidities, are presented in Table 1a. Metformin was the most reported antihyperglycemic medication, used by 164 (65.15%) participants. Participants also reported various non-diabetic chronic medical conditions. The most common were hyperlipidemia in 113 (44.8%), AH in 112 (44.4%), and CVD in 64 (25.4%) participants.

Regarding the laboratory parameters, kidney function tests showed a median creatinine level of 81 µmol/L (69–96.75), median albuminuria of 5 mg/mmol (2–13), and median ACR of 0.75 mg/mmol (0.46–1.73). Using the CKD-EPI equation for eGFR, participants were categorized as follows: more than 90 mL/min/1.73 m^2^ (70 participants, 27.8%), 60–89 mL/min/1.73 m^2^ (135 participants, 53.6%), 45–59 mL/min/1.73 m^2^ (23 participants, 9.1%), 30–44 mL/min/1.73 m^2^ (21 participants, 8.3%), and 15–29 mL/min/1.73 m^2^ (3 participants, 1.2%). Additionally, ACR values were grouped into three categories: less than 3 mg/mmol (208 participants, 82.5%), 3–30 mg/mmol (33 participants, 13.1%), and more than 30 mg/mmol (11 participants, 4.4%). After a complete laboratory blood and urine analysis, it was found that 77 participants (30.5%) had KD according to predefined criteria. All biochemical parameters for all 252 study participants are detailed in Table 1b.

Considering the body composition and anthropometric measurements, it is important to highlight that the median BMI was 27.6 kg/m^2^, with an interquartile range from 24.2 to 31 kg/m^2^. The distribution of BMI categories (normal BMI less than 25, overweight BMI from 25 to less than 30, and obese BMI with a value of 30 or more) among participants shows that 38.9% were overweight, 31.2% were obese, and 30% had normal BMI. Table 2 presents all other detailed data on the anthropometric measurements and the body composition of all study participants.

**Figure 2:**
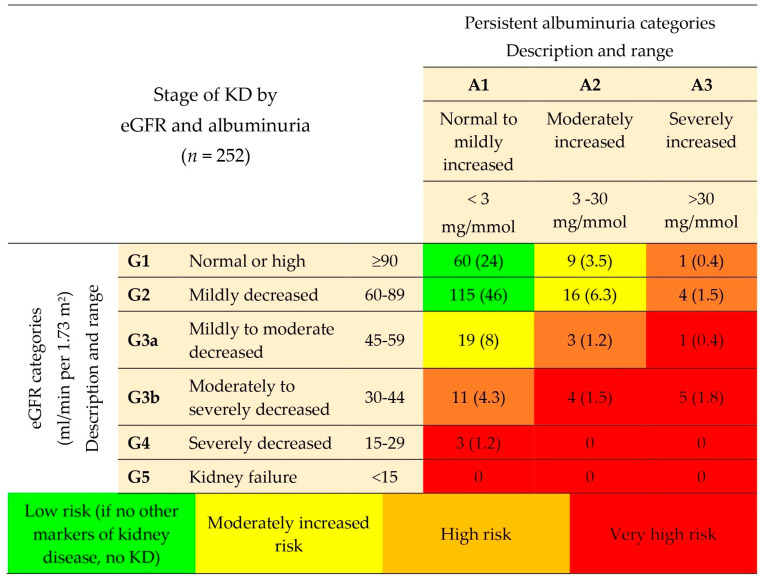
Stages of KD according to eGFR and albuminuria following KDIGO classification. Abbreviations: KD—kidney disease; eGFR—estimated glomerular filtration rate.

In a further analysis, only 6% of participants reported that they had KD, but after examining their lab results, 31% were found to have KD, highlighting a significant gap in awareness. Table 3 presents the discrepancy between participants’ self-reported knowledge of KD and the actual presence of KD as determined by laboratory results. Furthermore, Figure 2 depicts the stages of KD found in these participants according to KDIGO classification [27].

**Figure 3:**
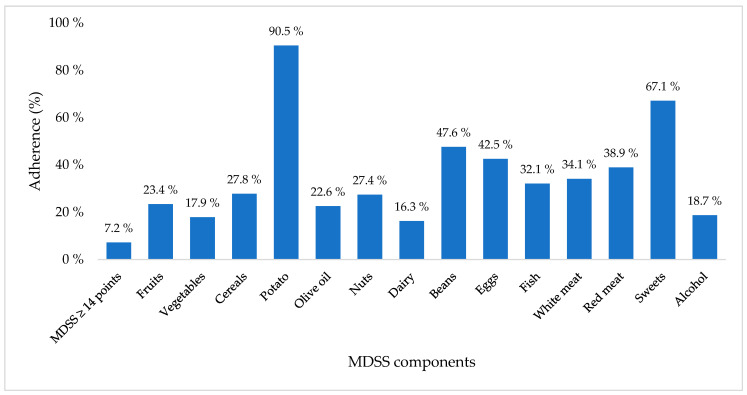
Adherence of all participants to the components of the Mediterranean Diet Serving Score. Abbreviations: MDSS—Mediterranean Diet Serving Score.

The adherence of all participants to the components of the MDSS is illustrated in Figure 3.

**Figure 4:**
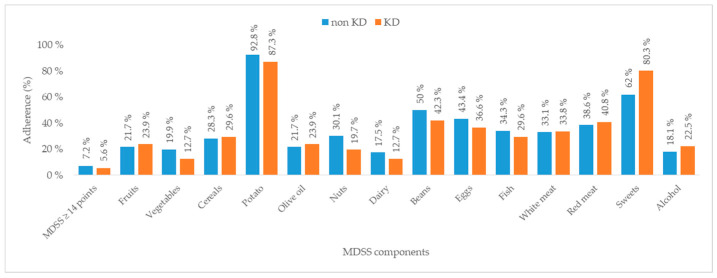
Adherence to the components of the Mediterranean Diet Serving Score based on the presence of kidney disease. Abbreviations: KD—kidney disease; MDSS—Mediterranean Diet Serving Score.

Participants were further categorized into two groups based on the presence of KD: non-KD and KD groups (Figure 4). When analyzing each dietary component, no significant differences were found between the groups, except in adherence to guidelines on sweets consumption (including sugar, cakes, cookies, fruit juices, and sodas). Specifically, 80.3% of participants with KD adhered to these guidelines compared to only 62% of non-KD participants (*p* = 0.006). Overall, there was no significant difference in total MDSS scores between the KD and non-KD groups (*p* = 0.58).

When examining the relationship between adherence to the MeDi and BMI, it was found that guidelines on nut consumption were most followed by participants with a normal BMI. Specifically, 37.8% of participants with a BMI < 25 kg/m^2^ adhered to these guidelines, compared to 27.1% of those with a BMI in overweight category, 18.2% of those with a BMI > 30 kg/m^2^, and 27.5% of all participants (*p* = 0.03). Additionally, obese participants (BMI > 30 kg/m^2^) most adhered to fresh fruit guidelines, excluding fruit juices. In this group, 31.2% of participants followed these guidelines, compared to 24.5% of those with a BMI < 25 kg/m^2^, 15.6% of those with a BMI in overweight category, and 23.1% of all participants (*p* = 0.05). All data are shown in Supplementary Table S1.

Using the Spearman correlation coefficient, statistically significant negative correlation was identified between eGFR and the duration of T2DM (Rho = −0.143, *p* = 0.02), and eGFR and pSBP (Rho = −0.153, *p* = 0.02), while no other statistically significant correlations were found, as presented in Table 4.

Additionally, laboratory results showed a negative correlation between MCV and MDSS (Rho = −0.139, *p* = 0.03), MCV and eGFR (Rho = −0.189, *p* < 0.001), and MCV and BMI (Rho = −0.153, *p* = 0.02). A statistically significant positive correlation was found between eGFR and RBC, and Hgb (Rho = 0.242, *p* < 0.001; and Rho = 0.161, *p* = 0.01, respectively). On the other hand, a negative correlation was observed between eGFR and neutrophils (Rho = −0.168, *p* = 0.01) and blood glucose levels (Rho = −0.124, *p* = 0.05). In analyzing correlations between BMI and lipid levels, a significant positive correlation was found with triglycerides (Rho = 0.346, *p* < 0.001), while a negative correlation was found with HDL (Rho = −0.331, *p* < 0.001). The complete correlations data discussed above are presented in Supplementary Table S2.

Logistic regression was used to test the influence of various predictors on the likelihood of increased KD risk. Six predictors were tested, revealing that younger age was associated with a lower likelihood of developing KD (OR = 0.95). Conversely, consumption of sweets, as well as higher levels of neutrophils, creatinine, triglycerides, and HDL, were associated with a higher likelihood of KD, with odds ratios of 2.57 (95% CI 0.922–0.997), 1.65 (95% CI 1.04–6.35), 1.08 (95% CI 1.24–2.19), 1.70 (95% CI 1.07–2.71), and 6.63 (95% CI 1.96–22.45), respectively. Data are presented in Table 5.

## 4. Discussion

### 4.1. Low Awareness of Kidney Health in Diabetic Population

Our study revealed a significant gap in awareness of KD in T2DM participants. Only 6% of participants believed they had KD, although the laboratory results showed that 31% had KD. This discrepancy emphasizes the urgent need for improved screening and awareness, as it is known that KD is often asymptomatic [28]. These results are in line with previously published studies implicating KD being undiagnosed in late stages. Only 6% of participants of the NHANES study with KD reported being told that they had weak or failing kidneys, and even at CKD stage 4, less than half participants were aware of their CKD [29]. Early detection of KD in T2DM patients is of utmost importance, and raising awareness is of urgent need. Our findings also revealed important correlations between kidney function and hematological and metabolic markers. A positive correlation was found between eGFR and hemoglobin and RBC count which is consistent with the evidence on the importance of normal kidney function in erythropoiesis [30,31]. On the other hand, a negative correlation was identified between eGFR and neutrophil count, as well as glucose level. A further analysis showed an inverse correlation between eGFR and both the duration of T2DM and systolic blood pressure, reinforcing the fact that regulated AH and DM are important factors in slowing CKD progression [32,33].

Focus should be placed on maximizing therapy while treating AH in diabetic individuals with CKD, as this has been shown to have extra benefits in reducing proteinuria, preventing eGFR decline, or improving cardiovascular health [33]. Our results also suggest several biochemical factors associated with an increased risk of developing KD. Younger age was associated with a lower risk of KD, and this finding is self-explanatory. Meanwhile, higher levels of neutrophils, creatinine, triglycerides, and HDL cholesterol were linked to an increased KD risk. These results emphasize the importance of precise dyslipidemia monitoring in T2DM. It is consistent with previous evidence [34]. It is interesting to note that only 36.9% of the participants in our study were prescribed statins. When it comes to neutrophil count being associated with the risk of KD in our participants, the results are like the results of a study by Jin-Li Gao et al., who found elevated neutrophil-to-lymphocyte ratio in older patients with T2DM highly associated with an increased risk of CKD [35].

### 4.2. Dietary Habits, Anthropometrics, and Body Composition of Diabetic Type 2 Participants

Overall adherence to the MeDi was not-surprisingly low (only 7.2% participants), in general Dalmatian population including islands adherence was also low (22.9%) [21] while participants with DM and AH in Dalmatia showed MeDi adherence of 8.9% [36]. Gerić et. al. found level of education and income correlated to MeDi adherence in the Croatian population, but the same authors showed how the MeDi diet can be followed in an affordable way by making a 7-day meal plan, which requires an average of EUR 6.98 per day [37].

The DMT2 participants in our study largely adhered to the MeDi recommendations on the consumption of potatoes and sweets. This may be due to the general and cultorological perception that diabetics should minimize sugar consumption. However, our findings highlight the need for further education and public health initiatives to promote awareness of comprehensive healthy dietary patterns and lifestyle changes, which are essential for reducing health risks in this population. Note that 80.6% of participants had received some form of nutritional advice, with the majority (60%) receiving advice from physicians, while only 11% received advice from dietitians and 6% from nurses. These results highlight the limited involvement of nurses and dietitians in providing nutritional counseling to diabetic patients in Dalmatia, indicating the need for greater interdisciplinary integration in patient care.

Obesity continues to be a growing public health problem. In 2019, Croatia was one of the countries with the most overweight people in Europe [38]. In our study, 70% of participants were classified as overweight or obese based on their BMI, which underlines the urgent need for targeted measures to combat this problem in the population.

It may seem obvious that obesity has one simple effect: excessive weight gain. But the spark that ignites subsequent metabolic disorders—of which T2DM is unquestionably the most closely associated with obesity—is a gradually rising body weight. Obesity plays a major role in the development and progression of DM, leading to a reshaped metabolic microenvironment, reducing insulin signaling and increasing blood glucose levels due to excessive nutrient accumulation, inflammation, autophagy disruption, and energy imbalance. This is primarily due to adipose tissue expansion, reprogramming immunometabolism, and pancreas toxication [39].

Our results show the association between BMI and lipid levels—positive correlation with triglyceride levels and negative with HDL. Recent studies have shown the elevated risk of dyslipidemia in obese people [40]. When compared to BMI categories, obese diabetic participants were most adherent to the MeDi recommendations on fruit intake.

Fruit has been demonstrated in several studies to have pro-obesity effects, and a few processes are believed to influence obesity through fruit consumption. Specifically, the predominant direct element thought to be accountable is the fruit’s high content of simple carbohydrates. Fruit therefore stores simple sugars instead of fat; however, fructose can still be significant. Through de novo lipogenesis, high fructose concentrations in meals are directly linked to several metabolic diseases, most notably obesity. Furthermore, eating fruit in ways other than whole can raise calorie intake and favorably affect energy homeostasis, which will ultimately encourage obesity. Like how fruit and fruit products cause obesity, fruit may also have an impact on metabolically healthy obesity. Although several studies have demonstrated that fruit contributes to obesity, the published trials’ findings are questionable due to a few issues, including the participants’ self-reporting of their fruit intake and the absence of daily energy-intake data [41,42].

On the other hand, guidelines on nut consumption were most followed by participants with a normal BMI. Contrarily, the results of a recent review showed no effect of nut consumption on the parameters of obesity in the T2DM population [43].

Another important result found was that those diabetic participants with KD, compared to those without KD, adhered more to the sweets intake guidelines of the MeDi. These findings could potentially be attributed to increased patient consciousness and more rigid disease treatment in individuals with T2DM and KD and possibly more frequent encounters with medical professionals, resulting in more structured counseling. It is significant to note that the cross-sectional design of this study naturally restricts the ability to determine any causal relationship between the mentioned variables. Future longitudinal studies are needed to explore these relationships over time.

### 4.3. Pharmacotherapy

When it comes to antihyperglycemics, a majority of participants were treated with metformin (65.1%), followed by insulin (27.8%), inhibitors of dipeptidyl peptidase 4 (DPP4i) (23.8%), sodium–glucose transport protein 2 inhibitors (SGLT2i) (17.9%), sulfonamides (12.7%), thiazolidinediones (4.4%), and alpha glucosidase inhibitors (3.6%). Despite substantial evidence supporting the use of GLP-1 receptor agonists (GLP-1RAs) and SGLT2i in patients with T2DM, their prescription rates remain conspicuously low within this cohort.

GLP-1RA were prescribed to only 5.7% of participants, a notably inadequate figure given the cohort’s clinical characteristics. CVD was present in 25.4% of participants, CBD in 2.8%, peripheral arterial disease in 4.4%, and obesity in 31.2%. GLP-1RAs have demonstrated a significant reduction in MACE and possess strong anti-obesogenic properties, making them particularly suited for this high-risk population [44,45,46,47,48,49]. Furthermore, in Croatia, GLP-1RAs are fully reimbursed for overweight diabetic patients with CVD or CBD, suggesting that prescription rates should be significantly higher. Underutilization in such a high-risk population reflects a critical gap in the optimization of patient care [50].

Similarly, the prescription rate for SGLT2i, at 17.9%, is also concerningly low. SGLT-2i demonstrated cardiovascular and renal benefits, in addition to promoting weight loss. Empagliflozin specifically provides protection against MACE and CKD, while dapagliflozin has been shown to delay CKD progression [51,52,53,54,55,56]. Given that 18.6% of participants had an eGFR below 60 mL/min/1.73 m^2^, the low prescription rate is particularly problematic. This shortfall may be partially attributed to only partial reimbursement of these agents by the Croatian Health Insurance Fund [50], as well as lack of screening for KD in T2DM participants and inadequate nephrology follow-up—only 12.7% of participants had regular nephrology consultations—highlighting a potential lack of awareness regarding KD risk and progression. Addressing these prescription gaps requires a concerted effort from specialists, including nephrologists, cardiologists, and diabetologists, to enhance both patient and general practitioner education. Improved awareness of the cardio-renal and anti-obesogenic benefits of GLP-1RA and SGLT2i will likely increase their utilization, fostering more comprehensive management of T2DM. Optimal diabetes care must extend beyond glycemic control, integrating cardiovascular and renal risk reduction, while addressing obesity and minimizing hypoglycemia risk [57,58].

### 4.4. Limitations

The main limitation of our study arises from its cross-sectional design, which prohibits any causal conclusion. The limitation of our study regarding pharmacotherapy is that we did not consider combinations of drugs, doses of drugs, duration of treatment, or type of insulin prescribed, which can influence KD progression. Furthermore, as we could not with certainty confirm the diagnosis of CKD according to KDIGO guidelines [27], only possible CKD (referred to in the manuscript as KD) was determined. In prospective studies and follow-ups, exact measurements (albuminuria follow-up) are to be further evaluated. The self-reported data used in this study to assess lifestyle and food habits may contain biases that could have an impact on the accuracy of the relationships found.

Although the conclusions of the study provide insightful information about awareness of kidney disease and lifestyle factors in patients with T2DM in the Dalmatia region, it is possible that these results do not apply to other groups. In addition to possible genetic influences, the unique cultural, dietary, and socioeconomic characteristics of this region could cause the population to differ from that of other locations. Therefore, caution should be used when extrapolating these results to larger or more diverse populations. Further research in other areas or nations with different genetic and socioeconomic backgrounds is needed to confirm and build on these results. Lastly, yet equally significant while not entirely novel, our findings of 31% undiagnosed KD and just 7.2% adherence to the MeDi underscore the urgent need for targeted interventions.

## 5. Conclusions

There is a place and urgent need for improvement in diabetes care in Dalmatia, with the emphasis on the awareness of the importance of prevention and early detection of KD in T2DM patients. Also, efforts should be put into multidisciplinary approach, education, and motivation of medical staff on dietary advising of diabetics and raising public awareness on this topic.

## Figures and Tables

**Table 1:** (**a**) General characteristics of studied population. (**b**) Laboratory parameters of studied population.

**(a)**		
		**All Participants** **(*n* = 252)**
Age (years), median (IQR)		67 (60–73)
Sex, *n* (%)	Men	122 (48.4)
	Women	130 (51.6)
T2DM ^1^ duration (years), median (IQR)		10 (6–20)
pSBP ^1^ (mm/Hg), median (IQR)		138 (125–152)
pDBP ^1^ (mm/Hg), median (IQR)		81 (75–88)
Prior endocrinologist examination, *n* (%)		178 (70.6)
Prior nephrologist examination, *n* (%)		81 (32.1)
Regular check-ups at the nephrologist, *n* (%)		32 (12.7)
Given dietary guidelines, *n* (%)		203 (80.6)
Self-reported adherence to dietary guidelines, *n* (%)		134 (53.2)
Dietary guidelines received by the following, *n* (%)	Physician	150 (59.5)
	Dietitian	28 (11.1)
	Nurse	16 (6.3)
	Other	10 (4)
MDSS ^1^, median (IQR)		7 (5–10)
Smoking, *n* (%)		44 (17.5)
Duration of smoking (years), median (IQR)		30 (25–44)
Former smokers, *n* (%)		87 (34.5)
Pharmacotherapy		
Insulin, *n* (%)		70 (27.8)
Metformin, *n* (%)		164 (65.1)
SGLT2i ^1^, *n* (%)		45 (17.9)
GLP-1RA ^1^, *n* (%)		14 (5.6)
DPP4i ^1^, *n* (%)		60 (23.8)
Sulfonamides, *n* (%)		32 (12.7)
Thiazolidinediones, *n* (%)		11 (4.4)
Alpha glucosidase inhibitors, *n* (%)		9 (3.6)
Statin, *n* (%)		93 (36.9)
Type of statin, *n* (%) (*n* = 86)	Atorvastatin	51 (59.3)
	Rosuvastatin	31 (36)
	Simvastatin	4 (4.7)
Prior chronic disease (self-reported)		
KD ^1^ per anamnesis, *n* (%)		16 (6.3)
KD ^1^ per laboratory results, *n* (%)		77 (30.5)
AH ^1^, *n* (%)		112 (44.4)
Hyperlipidemia, *n* (%)		113 (44.8)
Inflammatory rheumatic disease, *n* (%)		38 (15.1)
CVD ^1^, *n* (%)		64 (25.4)
CBD ^1^, *n* (%)		7 (2.8)
Malignancies, *n* (%)		18 (7.1)
PAD ^1^, *n* (%)		11 (4.4)
Depression, *n* (%)		8 (3.2)
Hypothyroidism, *n* (%)		22 (8.7)
(**b**)		
**Biochemical Parameter**		**All Participants** **(*n* = 252)**
Leukocytes (×10^9^/L), median (IQR)		6.8 (5.8–8.1)
RBC ^1^ (×10^12^/L), median (IQR)		4.7 (4.37–4.99)
Hb ^1^ (g/L), median (IQR)		141 (128–151)
MCV ^1^ (fL), median (IQR)		88.35 (86–91)
Neutrophile granulocytes (%), median (IQR)		59.25 (52.95–65.13)
Neutrophiles (×10^9^/L), median (IQR)		3.96 (3.13–5.12)
Lymphocytes (×10^9^/L), median (IQR)		2.03 (1.66–2.41)
Monocytes (×10^9^/L), median (IQR)		0.52 (0.42–0.65)
Glucose (mmol/L), median (IQR)		6.9 (5.7–9.08)
Creatinine (mmol/L), median (IQR)		81 (69–96.75)
eGFR ^1^ (mL/min/1.73 m^2^), median (IQR)		76.95 (62.48–91.18)
Cholesterol (mmol/L), median (IQR)		4.6 (3.8–5.48)
Triglycerides (mmol/L), median (IQR)		1.4 (0.9–2)
HDL^1^ cholesterol (mmol/L), median (IQR)		1.4 (1.2–1.7)
LDL^1^ cholesterol (mmol/L), median (IQR)		2.4 (1.8–3.1)
HbA1c ^1^ (%), median (IQR)		6.7 (6.1–7.3)
Albuminuria (mg/mmol), median (IQR)		5 (2–13)
ACR ^1^ (mg/mmol), median (IQR)		0.75 (0.46–1.73)
eGFR ^1^ (mL/min/1.73 m^2^), *n* (%)	≥90	70 (27.8)
	60–89	135 (53.6)
	45–59	23 (9.1)
	30–44	21 (8.3)
	15–29	3 (1.2)
ACR ^1^ (mg/mmol), *n* (%)	<3 mg/mmol	208 (82.5)
	3–30 mg/mmol	33 (13.1)
	>30 mg/mmol	11 (4.4)

^1^ Abbreviations: T2DM—type 2 diabetes mellitus; pSBP—peripheral systolic blood pressure; pDBP—peripheral diastolic blood pressure; MDSS—Mediterranean Diet Serving Score; SGLT2i—sodium–glucose transport protein 2 inhibitors; GLP-1RA—glucagon-like peptide-1 receptor agonist; DPP4i—inhibitors of dipeptidyl peptidase 4; KD—kidney disease; AH—arterial hypertension; CVD—cardiovascular disease; CBD—cerebrovascular disease; PAD—peripheral arterial disease. RBC—red blood cell count; Hb—hemoglobin; MCV—mean corpuscular volume; eGFR—estimated glomerular filtration rate; HDL—high-density lipoprotein; LDL—low-density lipoprotein; HbA1c—hemoglobin A1c; ACR—albumin-to-creatinine ratio, IQR-interquartile range.

**Table 2:** Anthropometric measurements of studied population.

Anthropometric Measures ^1^		All Participants (*n* = 252)
Hight (cm), median (IQR)		173 (165–180)
Body mass (kg), median (IQR)		81.5 (69.8–94.9)
BMI (kg/m^2^), median (IQR)		27.6 (24.2–31)
BMI categories, *n* (%)	BMI < 25	74 (30)
	25 ≤ BMI < 30	96 (38.9)
	BMI ≥ 30	77 (31.2)
MUAC (cm), median (IQR)		30 (27–32)
WC (cm), median (IQR)		100 (90–110)
HC (cm), median (IQR)		107 (100–114)
WHR, median (IQR)		0.93 (0.88–0.99)
FM (%), median (IQR)		30.1 (23.1–36.5)
FM (kg), median (IQR)		23.6 (17.9–31.8)
VF level, median (IQR)		11 (9–14)
FFM (kg), median (IQR)		56.8 (48.4–68.2)
PMM (%), median (IQR)		53.9 (45.9–64.8)
PhA (◦), median (IQR)		5.4 (4.8–5.9)

^1^ Abbreviations: BMI—body mass index; MUAC—mid-upper arm circumference; WC—waist circumference; HC—hip circumference; WHR—waist-to-hip ratio; FM—fat mass; VF—visceral fat; FFM—fat free mass; PMM—percentage of muscle mass; PhA—phase angle, IQR-interquartile range.

**Table 3:** Self-assessment of kidney disease versus kidney disease by laboratory results.

		Number (%) of Participants According to Kidney Disease Diagnosis			p *
		No	Yes	Total	
**Self-assessment of kidney disease diagnosis**	**No**	170	66	236 (94)	<0.001
	**Yes**	5	11	16 (6)	
	**Total**	175 (69)	77 (31)	252 (100)	

* McNemar–Bowker test.

**Table 4:** Correlation of age, duration of type 2 diabetes mellitus and blood pressure parameters with Mediterranean Diet Serving Score, estimated glomerular filtration rate, and body mass index.

	MDSS ^1,^*	eGFR ^1,^*	BMI ^1,^*
Age	0.031 (0.62)	−0.026 (0.68)	0.059 (0.35)
Duration of T2DM ^1^	−0.061 (0.34)	−0.143 (0.02)	−0.097 (0.13)
pSBP ^1^	−0.019 (0.77)	−0.153 (0.02)	0.125 (0.05)
pDBP ^1^	−0.028 (0.66)	0.108 (0.09)	0.112 (0.08)

* Data format: Rho (*p*-value). ^1^ Abbreviations: MDSS—Mediterranean Diet Serving Score; eGFR—estimated glomerular filtration rate; BMI—body mass index; T2DM—diabetes mellitus; pSBP—peripheral systolic blood pressure; pDBP—peripheral diastolic blood pressure.

**Table 5:** The influence of various predictors on the likelihood of increased kidney disease risk.

	ß	*p*	OR ^1^	95% CI ^1^
Age	−0.04	0.03	0.96	0.922–0.997
Consumption of sweets	0.95	0.04	2.57	1.04–6.35
Neutrophiles	0.40	0.002	1.49	1.15–1.93
Creatinine	0.07	<0.001	1.08	1.05–1.10
Triglycerides	0.41	0.04	1.51	1.01–2.27
HDL ^1^ cholesterol	1.74	0.002	5.72	1.95–16.8

^1^ Abbreviations: ß—regression coefficients; OR—odds ratio; CI—confidence interval; HDL—high-density lipoprotein.

## Data Availability

Data are available upon reasonable request made to the corresponding author via e-mail.

## Supplementary Materials

The following supporting information can be downloaded at https://www.mdpi.com/article/10.3390/medicina60101643/s1, Table S1: Differences in adherence to the Mediterranean Diet Serving Score (total and all components adherence) between three groups of participants among body mass index categories; Table S2: Correlation of laboratory results with Mediterranean Diet Serving Score, estimated glomerular filtration rate and body mass index.
