# Supplementary material for: Beyond Blood Sugar: Low Awareness of Kidney Disease among Type 2 Diabetes Mellitus Patients in Dalmatia—Insights from the First Open Public Call
Source: Medicina (Kaunas). 2024 Oct 8;60(10):1643. doi: 10.3390/medicina60101643 (PMC11509393; doi:10.3390/medicina60101643)
Supplement: Supplementary file 1 [file medicina-60-01643-s001.zip › medicina-3223607-supplementary.pdf]

Supplementary Table S1. Differences in adherence to the Mediterranean Diet Serving Score (total and all components adherence) between three groups of participants among body mass index categories.

	Number of participants (%)				
	**Normal ($< 25 kg/m^2$)**	**Overweight ($25 \leq BMI < 30$);**	**Obese ($\geq 30 kg/m^2$)**	**Total**	***P****
Cereals	21 (28.4)	25 (26)	22 (28.6)	68 (27.5)	0.92
Potato	64 (86.5)	87 (90.6)	72 (93.5)	223 (90.3)	0.34
Olive oil	16 (21.6)	21 (21.9)	19 (24.7)	56 (22.7)	0.88
Nuts	28 (37.8)	26 (27.1)	14 (18.2)	68 (27.5)	0.03
Fresh fruit	18 (24.3)	15 (15.6)	24 (31.2)	57 (23.1)	0.05
Vegetables	14 (18.9)	14 (14.6)	14 (18.2)	42 (17)	0.72
Milk and dairy products	13 (17.6)	13 (13.5)	14 (18.2)	40 (16.2)	0.66
Legumes	34 (45.9)	46 (47.9)	38 (49.4)	118 (47.8)	0.92
Eggs	34 (45.9)	40 (41.7)	29 (37.7)	103 (41.7)	0.59
Fish	21 (28.4)	34 (35.4)	25 (32.5)	80 (32.4)	0.62
White meat	31 (41.9)	31 (32.3)	22 (28.6)	84 (34)	0.20
Red meat	30 (40.5)	35 (36.5)	31 (40.3)	96 (38.9)	0.83
Sweets	50 (67.6)	66 (68.8)	49 (63.6)	165 (66.8)	0.77
Wine	8 (10.8)	22 (22.9)	17 (22.1)	47 (19)	0.09
MDSS total, median (IQR)	6 (5 – 10)	6 (4 – 9)	7 (4 – 11)	7 (5 – 10)	0.53†

* χ^2 test; † Kruskal Wallis test

[1] Abbreviations: BMI – body mass indeks, MDSS – Mediterranean Diet Serving Score.

Supplementary Table S2. Correlation of laboratory results with Mediterranean Diet Serving Score, estimated glomerular filtration rate and body mass index.

**Biochemical parameter**[1]	**MDSS**[1]	**eGFR**[1]	**BMI**[1]
WBC	-0.111 (0.08)	0.009 (0.89)	0.154 (0.02)
RBC	0.091 (0.15)	0.242 (< 0.001)	0.161 (0.01)
Hb	-0.016 (0.81)	0-161 (0.01)	0.103 (0.11)
MCV	-0.139 (0.03)	-0.189 (< 0.001)	-0.153 (0.02)
MCH	-0.157 (0.01)	-0.079 (0.21)	-0.103 (0.11)
MCHC	-0.083 (0.19)	0.130 (0.04)	0.038 (0.55)
Neutrophile granulocytes	-0.033 (0.60)	-0.168 (0.01)	0.024 (0.70)
Lymphocytes	0.071 (0.26)	0.190 (< 0.001)	-0.045 (0.49)
Neutrophiles	-0.103 (0.10)	-0.072 (0.25)	0.098 (0.12)
Lymphocyte	-0.040 (0.53)	0.162 (0.01)	0.119 (0.06)
Monocytes	-0.157 (0.01)	-0.045 (0.48)	0.112 (0.08)
Glucose	0.027 (0.67)	-0.124 (0.05)	-0.001 (0.99)
Creatinine	-0.025 (0.70)	-0.795 (< 0.001)	0.013 (0.84)
eGFR	-0.019 (0.76)	/	0.013 (0.84)
Total cholesterol	0.017 (0.79)	0.091 (0.15)	0.006 (0.93)
Triglycerides	0.025 (0.69)	-0.097 (0.12)	0.346 (< 0.001)
HDL cholesterol	-0.018 (0.77)	0.085 (0.18)	-0.331 (< 0.001)
LDL cholesterol	0.001 (0.98)	0.103 (0.10)	0.012 (0.85)
HbA1c	0.024 (0.71)	-0.042 (0.50)	-0.074 (0.25)
Albuminuria	0.048 (0.45)	-0.017 (0.78)	0.108 (0.09)

[1] Abbreviations: MDSS – Mediterranean Diet Serving Score, eGFR – estimated glomerular filtration rate, BMI – body mass indeks, WBC – white blood cell count, RBC – red blood cell count, Hb – hemoglobin, MCV – mean corpuscular volume, MCH – mean celullar hemoglobin, MCHC – mean cellular hemoglobin concentration, HDL – high density lipoprotein, LDL – low density lipoprotein, HbA1c – hemoglobin A1c, ACR - albumin-to-creatinine ratio.
